# Microbial Tryptophan Metabolism Tunes Host Immunity, Metabolism, and Extraintestinal Disorders

**DOI:** 10.3390/metabo12090834

**Published:** 2022-09-03

**Authors:** Moyan Liu, Max Nieuwdorp, Willem M. de Vos, Elena Rampanelli

**Affiliations:** 1 Department Experimental Vascular Medicine, Amsterdam University Medical Center (AUMC, Location AMC), Amsterdam Gastroenterology Endocrinology Metabolism (AGEM) Research Institute, Amsterdam Cardiovascular Science (ACS) Institute, 1105AZ Amsterdam, The Netherlands; 2 Department (Experimental and Clinical) Vascular Medicine, Amsterdam University Medical Center (AUMC, Location AMC), Amsterdam Diabetes Center, Amsterdam Gastroenterology Endocrinology Metabolism (AGEM) Research Institute, Amsterdam Cardiovascular Science (ACS) Institute, 1105AZ Amsterdam, The Netherlands; 3 Laboratory of Microbiology, Wageningen University, 6700EH Wageningen, The Netherlands; 4 Human Microbiome Research Program, Faculty of Medicine, University of Helsinki, 00014 Helsinki, Finland

**Keywords:** tryptophan, microbiota, inflammation, metabolic syndrome, chronic kidney diseases (CKD), cardiovascular diseases (CVD)

## Abstract

The trillions of commensal microorganisms comprising the gut microbiota have received growing attention owing to their impact on host physiology. Recent advances in our understandings of the host–microbiota crosstalk support a pivotal role of microbiota-derived metabolites in various physiological processes, as they serve as messengers in the complex dialogue between commensals and host immune and endocrine cells. In this review, we highlight the importance of tryptophan-derived metabolites in host physiology, and summarize the recent findings on the role of tryptophan catabolites in preserving intestinal homeostasis and fine-tuning immune and metabolic responses. Furthermore, we discuss the latest evidence on the effects of microbial tryptophan catabolites, describe their mechanisms of action, and discuss how perturbations of microbial tryptophan metabolism may affect the course of intestinal and extraintestinal disorders, including inflammatory bowel diseases, metabolic disorders, chronic kidney diseases, and cardiovascular diseases.

## 1. Introduction

The trillions of microbes that inhabit the human gastrointestinal tract have evolved an intimate and mutualistic relationship with their host. The collection of gut microorganisms outnumbers the host in terms of cell numbers as well as genetic composition, with more than 22 million non-redundant genes identified in the gut [1,2]—a staggering number when considering that the human genome harbors 20,000–25,000 genes [3]. Unsurprisingly, this vast and diverse microbial ecosystem plays a vital role in the nutrition and physiology of the host, exerting metabolic functions such as the degradation of complex carbohydrates into short-chain fatty acids (SCFAs), biosynthesis of vitamins, generation of secondary bile acids, and stimulating the development and function of the immune system [4,5]. A pivotal component of microbiota–host interactions is the microbial production of a wide array of small-molecule metabolites, which are either synthesized de novo or metabolized from dietary nutrients or host compounds [6]. These microbiota-derived metabolites act as signaling molecules or metabolic precursors not only in the intestinal milieu, but also in distal organs, following absorption into the circulation [4,7].

Diet, geography, ethnicity, age, and genetics dictate the taxonomic profile of the gut microbiome and, consequently, the commensal metabolic capacities [8,9,10,11,12,13,14,15]. Perturbations in the composition and metabolic functions of the gut microbiota are associated with a myriad of inflammatory and chronic metabolic diseases, including inflammatory bowel diseases (IBDs), autoimmunity, diabetes, obesity, chronic kidney diseases (CKDs), and cardiovascular diseases (CVDs) [4,5,7].

**Figure 1:**
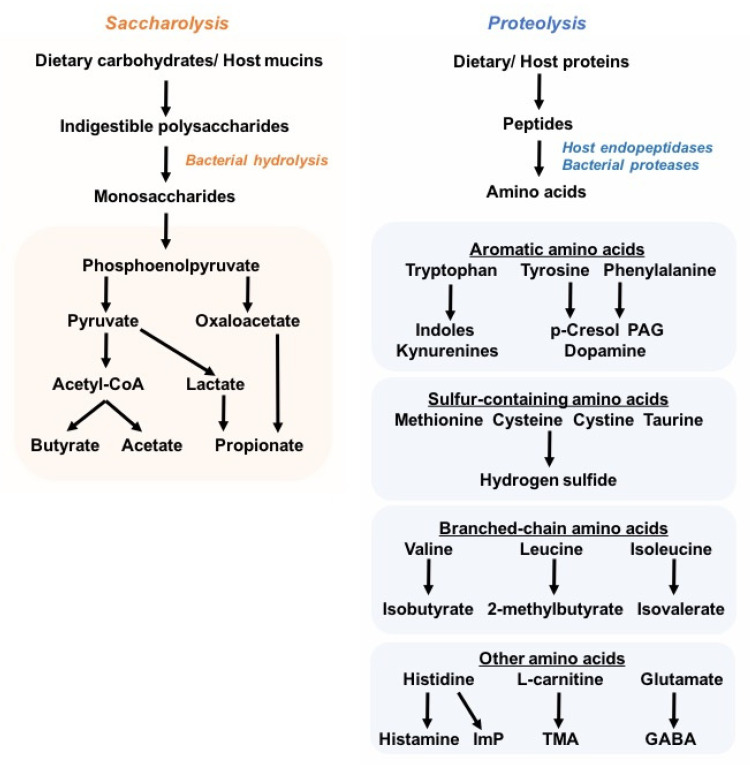
Myriad commensal bacteria can metabolize dietary and host-derived carbohydrates or proteins generating short-chain fatty acids (e.g., butyrate, acetate, propionate, and lactate) and diverse amino acids’ catabolites (through diverse bacterial pathways degrading aromatic, sulfur-containing, branched-chain, and other amino acids), respectively.

In healthy adults, the gut microbiome comprises five main phyla: *Firmicutes*, *Bacteroides*, *Proteobacteria, Actinobacteria*, and *Verrucomicrobia*, although *Firmicutes* (60–80%) and *Bacteroides* (15–25%) are the most abundant [16]. The introduction of new high-throughput molecular sequencing technologies such as 16S ribosomal RNA (rRNA) gene sequencing and whole-metagenome shotgun sequencing, as well as untargeted metabolomics approaches, has greatly advanced the microbiota research field, and helps in depicting the taxonomic composition and, moreover, the functional genomic and metabolic capacity of commensal bacterial communities. Due to the large interindividual variation in the consortium of microbial species in the human gut, a universal definition of a healthy microbiome remains challenging; nonetheless, microbial diversity with notably high species richness is postulated to be main trait of a healthy microbiome, together with the ability to live in an immunotolerant environment in the host gastrointestinal tract. The high species richness is thought to render the overall microbial community more stable and resilient to environment- and host-derived perturbations, and is partially reflected by the myriad of microbial metabolites that constitute the intestinal milieu and contribute to the pool of circulating metabolites [6,17]. Unsurprisingly, studies have shown that disease-associated microbial signatures are correlated with changes in plasma metabolome and microbial metabolite levels. In particular, the imbalance in protective and deleterious microbial metabolites is believed to drive disease progression and severity [18,19,20,21,22]. Despite advances in metagenomic and metabolomic approaches as well as culture techniques to characterize the microbiota, only a handful of microbiota-derived byproducts have been identified for their specific roles in health and diseases (Figure 1). Particularly, the SCFAs butyrate, propionate, and acetate are generated by the bacterial degradation of dietary fibers, and have been extensively studied owing to their health-promoting effects against inflammatory and metabolic disorders, such as colitis and obesity [23]. In contrast to saccharolytic products, proteolysis-derived microbial metabolites have received less attention, and are largely considered to be deleterious to the host’s (patho)physiology, e.g., trimethylamine N-oxide (TMAO) [24,25,26], imidazole propionate [19,27], phenylacetylglutamine (PAG) [28,29,30], p-cresol sulfate (PCS), and indoxyl sulfate (IS) [31,32,33]. Nonetheless, lysine, which is the most abundant amino acid in foods, can be converted into the beneficial butyrate by bacteria related to *Intestinimonas* spp. [34].

Among these bacterial proteolytic byproducts, the microbial production of tryptophan metabolites has received increasing attention owing to the multifaceted impacts of tryptophan derivatives on several aspects of host physiology, along with the versatility of their functions, which appear to be cell-/tissue-specific and disease-dependent. In this review, we summarize and discuss recent findings related to the proteolytic degradation of the amino acid tryptophan by gut microbes, and to the roles of these tryptophan catabolites in health and disease.

## 2. Analytical Approaches Aiding Microbiota–Metabolome Studies

Metabolomic approaches are rapidly developing, and substantially support host–microbiome studies, particularly when metabolic analyses are coupled with enzymatic activity assays and in vitro cultures of single strains or consortia of gut bacteria [35]. Mass spectrometry has become a pivotal tool in the characterization of metabolite profiles in distinct host compartments, such as peripheral blood, portal vein blood, and stool and tissue biopsies, owing to its high-sensitivity, high-throughput features and ability to analyze large numbers of samples. In particular, liquid chromatography–mass spectrometry (LC–MS) is widely used as an analytical tool for its applicability to a vast array of metabolites, including polar and non-polar metabolite classes, e.g., amino acids (including tryptophan and its derivatives), sugar metabolites, vitamins, lipid, fatty acids, and bile acids [36,37,38,39]. Conversely, gas chromatography–mass spectrometry (GC–MS) is more applicable for volatile metabolites, such as SCFAs, although it can also be used to analyze sugars, amino acids, and related compounds [40]. Untargeted metabolomics is particularly useful for screening a wide array of metabolite classes and, combined with fecal/small intestine microbiota analysis, can determine important bacteria–metabolite associations and identify putative microbial metabolites linked to specific disease characteristics. Although ideal for unbiased discovery approaches, untargeted metabolomics can determine changes in metabolite abundance relative to controls or baseline, but does not provide actual concentrations of specific metabolites, due to limited usage of molecular standards. In addition, MS spectra are not fully annotated in databases, resulting in a majority of undefined compounds during unbiased profiling. In contrast, targeted metabolic analysis, which should always be used to validate metabolite discovery by untargeted MS, enables absolute compound quantification and optimized preprocessing and extraction methods for a specific metabolite class [41]. Notably, in addition to determining the physiological concentrations of metabolites in specific host compartments, it is crucial to establish whether newly discovered metabolite–microbiota or metabolite–disease associations are found in different patient cohorts—especially in light of the high inter- and intraindividual variability of both microbiome and metabolome profiles. Importantly, MS methods also allow for the quantification of isotopic labelling, enabling the analysis of metabolic fluxes in bacteria from labelled dietary/host products; however, this approach remains challenging in complex microbial communities in vivo, as bacteria may share and cross-feed one another’s metabolite products.

The identification of disease-relevant gut metabolites requires well-controlled cross-sectional studies and well-phenotyped longitudinal patient cohorts to adjust for the effects of gut microbial modifiers—such as age, diet, ethnicity, sex, and smoking—minimize the interindividual variability, and study changes in metabolic signatures over time with bacterial abundancy and metabolic capacity, as well as with the course of the disease. Nonetheless, to unveil the role of putative microbial metabolites in disease severity/onset, and to exclude host origin, the use of animal models—particularly gnotobiotic mice—remains the gold standard in microbiome research to prove causality [41,42].

## 3. Tryptophan Catabolism

Although most dietary proteins are absorbed in the small intestine, a relevant proportion of dietary and host proteins (estimated 5–18 g/day) reaches the colon, where proteins are degraded by both host endopeptidases and bacterial proteases [43,44,45]. The resulting amino acids are further digested and deaminated through bacterial fermentation. Notably, the aromatic amino acids may generate indole and its derivatives, as well as phenolic compounds that may be undesired. Protein fermentation by colonic microbes also contributes to a small proportion of the total microbiota-derived SCFAs (e.g., acetate, propionate, and butyrate), while the branched-chain amino acids give rise to the branched-chain FAs (e.g., isobutyrate, 2-methylbutyrate, isovalerate) [46]. 

Microbial protein fermentation is favored by low carbohydrate availability, high dietary protein intake, increased colonic pH, and prolonged colonic transit time [47,48,49,50]. In cases of fiber depletion, the bacterial shift from saccharolytic toward proteolytic metabolism results in lower production of beneficial SCFAs; these perturbations in bacterial metabolic activities and the constriction of saccharolytic bacteria are often associated with diseased states and gut-dysbiotic signatures [4,5,7,18,19,51,52,53,54,55]. However, in the case of tryptophan catabolism, the products of its degradation have been linked to both health benefits and poor health outcomes [7,56,57,58].

Tryptophan is one of the nine essential amino acids found in common protein-rich foods, such as milk, cheese, eggs, meat, fish, bananas, oats, nuts, and beans. Tryptophan is largely absorbed in the small intestine, and the fraction that reaches the colon is catabolized by numerous bacterial species, including *Escherichia coli*, *Clostridium*, *Bacteroides*, *Peptostreptococcus*, *Eubacterium*, and *Lactobacillus* species, and *Ruminococcus gnavus* [50,59,60,61,62,63,64,65,66,67]. In addition, the breakdown of tryptophan by bacteria is not exclusive to the distal colon, as lactobacilli have been reported to catabolize tryptophan in the stomach and small intestine [56]. 

**Figure 2:**
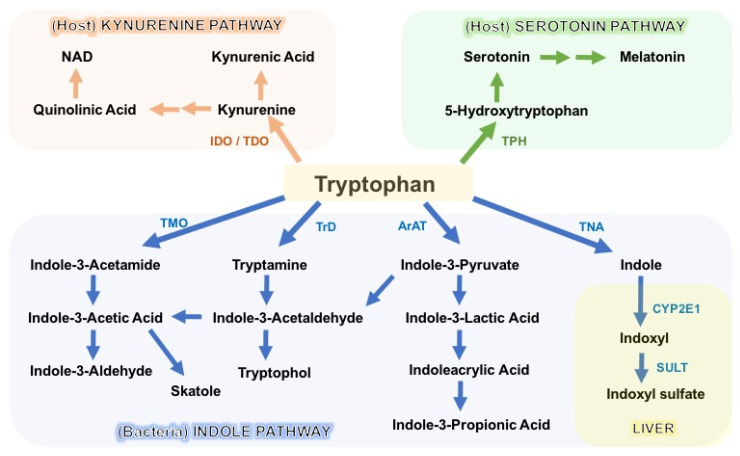
Simplified illustration of the eukaryotic and bacterial pathways of tryptophan degradation in the gastrointestinal tract: Eukaryotic enzymes: IDO (indoleamine 2,3-dioxygemase), TDO (tryptophan 2,3-dioxygemase), TPH (tryptophan hydroxylase), CYP2E1 (cytochrome P450 2E1), SULT (sulfotransferase). Bacterial enzymes: TMO (tryptophan 2-monooxygenase), TrD (tryptophan decarboxylase), ArAT (aromatic amino acid aminotransferase), TNA (tryptophanase), IDO homologs.

The degradation of tryptophan occurs through three major pathways: the kynurenine pathway (KP), the serotonin pathway, and the indole pathway (Figure 2). The KP is the primary degradation route in mammalian cells, and encompasses a series of enzymatic steps, with consequential formation of N-formylkynurenine, kynurenine (Kyn), 3-hydroxykynurenine (3-OHKyn), kynurenic acid (kna), 3-hydroxyanthranilic acid (3HAA), quinolinic acid and, ultimately, NAD^+^. The rate-limiting step in the KP is catalyzed by indoleamine 2,3-dioxygenase-1/2 (IDO1/2) or tryptophan 2,3-dioxygenase (TDO) [58,68]. Following enterocyte-mediated absorption, tryptophan is transported via the hepatic portal system to the liver for utilization by the intrahepatic TDO, whereas the remaining untouched tryptophan is secreted into the circulation for utilization by peripheral tissues. Here, the IDO1 enzyme (expressed in immune and intestinal epithelial cells) is the main driver of tryptophan degradation, as compared to the IDO2 isoform. IDO1 is not constitutively expressed, and it is upregulated in response to inflammation and indigenous gut bacteria. Conversely, IDO1 activity and downstream catabolite formation modulate mucosal reactivity and, hence, microbiota composition [68,69]. In addition to host generation of kynurenine, several intestinal bacteria encode homologous KP enzymes, and a few gut microbes (i.e., *Lactobacillus* spp., *Pseudomonas aeruginosa*, and *P. fluorescens*) have been shown to produce kynurenine derivatives [69,70]. Depending on the targeted tissue and environment, KP metabolites can either exert beneficial effects on host homeostasis or contribute to disease progression. For instance, they promote intestinal homeostasis, immunotolerance, energy expenditure, and resistance to stress-induced depression, but appear deleterious in the context of cancer, metabolic syndrome, and atherosclerosis [71,72,73,74,75,76,77]. 

Another important catabolite of ingested tryptophan is the neurotransmitter serotonin (5-hydroxytryptamine, 5-HT), which is mainly synthesized by intestinal enterochromaffin cells via tryptophan hydroxylase 1 (TPH1). Serotonin modulates several aspects of host physiology by stimulating (among others) intestinal peristalsis via 5-HT receptor signaling, vasodilatation, and platelet function [78]. A smaller portion of 5-HT is generated in the serotonergic neurons via the THP2 isoform of the enteric and central nervous system, where it modulates mood, appetite, sleep, and cognition [79]. Commensal microbes seem to regulate 5-HT production, since germ-free mice display impaired intestinal production and reduced circulating levels of serotonin [62,80]. Although the mechanistic evidence is limited, two studies showed that SCFAs induce the expression of *Tph1*, and that the bacterial secondary bile acid deoxycholate can restore the colonic and blood levels of 5-HT [81,82]. Moreover, analysis of fecal metagenomes to profile metabolic pathways for neurotransmitter synthesis revealed that gut bacteria have the genomic potential for 5-HT synthesis, although direct evidence of this process is still lacking [83]. Nonetheless, two commensal *Firmicutes* bacteria (*Clostridium sporogenes* and *Ruminococcus gnavus*) possess two phylogenetically distinct enzymes that decarboxylate tryptophan to generate tryptamine—a biogenic amine that stimulates the release of serotonin by enterochromaffin cells [67,84]. Moreover, tryptophan decarboxylase homologous genes were found in 9–17% of gut metagenomes of healthy humans—particularly in the genomes of other *Firmicutes*, suggesting that more commensal microbes may regulate the release of 5-HT [67]. 

Lastly, the gut microbes provide a third important route of tryptophan catabolism through the direct transformation of tryptophan into tryptamine and indole metabolites via the action of the bacterial enzymes decarboxylase and tryptophanase A (TnaA), respectively. As described above, bacterial tryptophan decarboxylases have been identified in two gut *Firmicutes*: *Clostridium sporogenes* and *Ruminococcus gnavus* [67]. Conversely, the enzyme TnaA is expressed by many Gram-negative and Gram-positive commensal bacteria. Many indigenous microbes have been shown to metabolize tryptophan into indole, including *Escherichia coli*, *Clostridium* spp., *Bacteroides* spp., *Lactobacillus* spp., and *Streptococcus* spp. [50,56,63,85,86,87]. In addition, gut microbes amplify the variety of tryptophan catabolites through oxidative and reductive pathways generating various indole derivatives. For instance, indole-3-pyruvic acid (IPYA) can be converted into indole-3-lactic acid (ILA), and successively into indole-3-propionic acid (IPA), or IPYA gives rise to indole-3-acetylaldehyde, which is further processed into indole-3-acetic acid (IAA) and, subsequently, into indole-3-aldehyde (IAld) [69] (Figure 2). Microbiota-produced indoles are detected in the circulation and feces at μM concentrations, and are excreted in the urine [88,89,90,91]. Once absorbed into the circulation, indole can be further converted in the liver into indoxyl sulfate, which has been implicated in the pathogenesis of chronic kidney diseases (CKDs) and cardiovascular comorbidities [7]. Despite these deleterious effects, most indole derivatives—such as indolelactic acid (ILA), IAA, IPA, and IAld—are key modulators of intestinal homeostasis, endorsing barrier integrity, epithelial renewal, and fine-tuning of mucosal immune responses [56,92,93,94]. 

In addition, indole and its derivatives act as interspecies signaling molecules in microbial communities by affecting sporulation, drug resistance, and biofilm formation. For instance, ILA has been reported to exert antifungal and antibacterial activities, whereas indole-ethanol (tryptophol) possesses antibacterial and antiphagic properties. Nonetheless, it remains to be elucidated whether these effects substantially modulate the gut microbial ecosystem [69,95,96,97].

**Figure 3:**
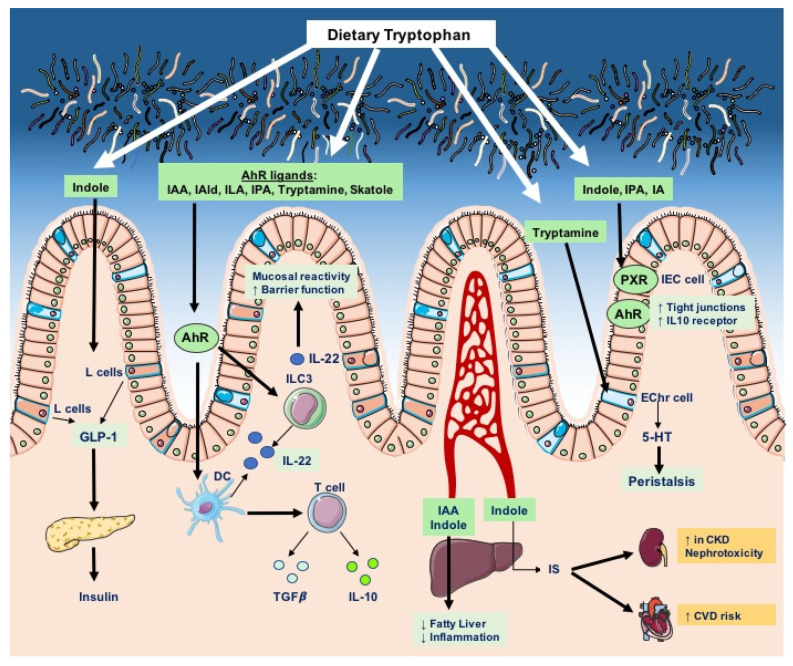
Microbiota-derived tryptophan metabolites modulate various host physiological processes. From left to right: indole signals to enteroendocrine L cells to induce the secretion of GLP-1, which stimulates insulin secretion by pancreatic beta cells. Many of the bacterial tryptophan catabolites—such as indole acetic acid (IAA), indole-3-acetaldehyde, indole-3-aldehyde (IAld), indole-3-lactic acid (ILA), indole-3-propionic acid (IPA), tryptamine, and skatole—activate the aryl hydrocarbon receptor (AhR) in intestinal immune cells; AhR activation in dendritic cells (DC) and type 3 innate lymphoid cells (ILC3) promotes production of IL-22—a central cytokine for intestinal homeostasis—and favors the expansion of anti-inflammatory T cells. After absorption into the bloodstream, indoles reach the liver, where they exhibit protective effects against lipid accumulation and inflammation; however, hepatic enzymes can convert indole into the uremic toxin indoxyl sulfate (IS), which is harmful to kidney epithelial cells as well as endothelial cells. IS levels are correlated with kidney dysfunction in chronic kidney diseases (CKDs) and increased risk of cardiovascular disease (CVD). Microbial tryptamine can incite enterochromaffin cells (EChr) to release 5-hydroxytryptamine (5-HT), which stimulates gastrointestinal mobility. Lastly, indoles regulate intestinal homeostasis and barrier function by activating AhR and the pregnane X receptor (PXR) within intestinal epithelial cells (IECs).

Overall, host and bacterial tryptophan degradation pathways are integral components of host physiology, as they give rise to a variety of bioactive molecules that regulate barrier function, metabolism, inflammation, and endocrine and neuronal activities (Figure 3). In the next sections, we discuss the implications of tryptophan metabolites—particularly of catabolites of microbial origin—in homeostatic and diseased conditions.

## 4. Tryptophan Catabolites as AhR Ligands: Role in Intestinal Homeostasis 

Numerous studies have underlined that microbiota-derived tryptophan metabolites contribute to intestinal homeostasis largely through activation of the aryl hydrocarbon receptor (AhR). AhR is a transcription factor kept inactive in a cytosolic multiprotein complex; upon ligand binding and activation, AhR translocates to the nucleus, where it drives the expression of target genes harboring xenobiotic response DNA elements (XREs). Notably, AhR senses both endogenous and exogenous factors, such as toxins, polyaromatic hydrocarbons, and microbial metabolites. Indeed, many microbial tryptophan derivatives—such as indole-3-acetic acid, indole-3-aldehyde, indole-3-acetaldehyde, indole-3-propionic acid, indole-3-lactic acid, indoleacrylic acid, tryptamine, 3-methylindole (skatole), and indoxyl sulfate—as well as bacteria- or host-derived kynurenine, are AhR agonists [58,69]. Upon ligand-mediated activation, AhR drives the expression of enzymes crucial for the metabolic detoxification of xenobiotics, such as cytochrome p450 (CYP1), as well as the upregulation of key enzymes (e.g., IDO1, TDO) of the kynurenine pathway. This indicates that microbiota-produced tryptophan catabolites indirectly control the rate of tryptophan degradation by the host. In addition to controlling gene expression, AhR modulates and integrates immune pathways at multiple levels; for example, AhR interacts with members (e.g., RelA, RelB) of the NF-κB complex and, along with its negative regulator SOCS2 (suppressor of cytokine signaling 2), it regulates the activity of the immune signal transducers STAT1 and STAT3, and limits the cellular levels and transcriptional activity of HIF-1α (hypoxia-inducible factor 1α) [98,99,100,101,102,103].

The AhR transcription factor is widely expressed by immune cells and intestinal epithelial cells (IECs; moreover, AhR signaling is a key component of intestinal homeostasis and the maintenance of host–microbiota symbiosis [69]. In mice, IEC-specific *AhR* deletion leads to aberrant inflammation and impairs IEC differentiation, rendering mice more susceptible to pathogenic infection due to a defective intestinal barrier and malignant transformation due to uncontrolled intestinal stem cell proliferation [104]. Similarly, specific ablation of AhR signaling in mucosal dendritic cells (DCs) impairs epithelial morphogenesis (with reduced Paneth cell differentiation and increased goblet cell differentiation) as well as barrier function, resulting in the exacerbation of inflammation in experimental colitis [105]. Conversely, intact AhR signaling downstream of tryptophan catabolism drives the expression of the interleukin-10 (IL-10) receptor on IECs, thereby promoting barrier function as well as epithelial wound healing. Furthermore, exogenous kynurenine was found to protect mice from chemically induced colitis [106]. Similarly, AhR activation can promote immunotolerance by inducing the generation of tissue-protective intraepithelial lymphocytes producing anti-inflammatory cytokines, such as IL-10 and transforming growth factor β (TGF-β) [107,108]. In DCs, the AhR-mediated upregulation of IDO1 results in the conversion of tryptophan into kynurenine, which reduces the immunogenicity of DCs and promotes the differentiation of immunoregulatory T(reg) cells [109,110,111]. 

In addition, AhR drives the development of intestinal IL-22-producing innate lymphoid cells (ILC3s), as well as the expression of IL-22 [112]. The latter is a crucial cytokine for intestinal homeostasis produced in the intestinal mucosa by DCs, CD4 T cells, and ILC3s [112,113]. Indeed, IL-22 exerts protection against pathogenic infections, fortifies the intestinal barrier by increasing the expression of tight-junction molecules, and limits mucosal inflammation in colitis [113,114]. Moreover, the AhR-driven IL-22 expression in ILC3s protects mice from enteric *Citrobacter rodentium* and *Toxoplasma gondii* infections, and restricts the colonization by commensal segmented filamentous bacteria (SFB), which are capable of specifically inducing T-helper 17 (Th17) cells in the gut [112,115,116,117,118]. Although SFB are generally not present in the human gut, these findings translate to human inflammatory bowel disease (IBD), where AhR expression is significantly downregulated and ILC1s accumulate in the inflamed ileum of patients with Crohn’s disease, at the expense of ILC3s [114,119]. Accordingly, a metagenomics analysis of stool samples from IBD patients revealed a reduced genomic capacity of gut microbes to metabolize tryptophan [86]. Similarly, the microbiota of individuals with IBD displayed an impaired capacity to produce AhR ligands, together with diminished levels of tryptophan and IAA [120]. Compared to healthy controls, IBD patients display reduced levels of serum tryptophan but higher kynurenine/tryptophan ratios, together with an enhanced expression of *IDO1* in colonic biopsies; furthermore, disease activity has been found to be inversely correlated with serum tryptophan levels [121,122]. These findings suggest that in IBD the increased tryptophan catabolism in gut immune cells—associated with impairment in the microbial capacity to degrade tryptophan—may limit the bioavailability of beneficial indole derivatives and contribute to disease severity.

Finally, intestinal AhR signaling impacts the gut microbiota composition; indeed, mice fed with an AhR-ligand-free diet or supplemented with the agonist indole-3-carbinol have a different fecal microbiota profile. In fact, an AhR-ligand-free diet causes enrichment in the family Erysipelotrichaceae and a reduction in fecal levels of immunoglobulin A (IgA), which is pivotal in the fight against enteric infections, but also for the proper containment of indigenous bacteria and maintenance of immune homeostasis [4,123,124].

Overall, these studies underline a physiological role of AhR activation in fine-tuning the intestinal inflammatory tone [107], as well as the morphology and function of the intestinal epithelial layer—all of which are phenomena that can impact the intestinal microbial ecosystem. Likewise, the microbial communities can affect host cellular interactions and signaling by releasing a myriad of metabolites. In the next section, we focus on the role of microbiota-produced tryptophan metabolites in intestinal wellbeing and inflammatory conditions.

## 5. Tryptophan Catabolites in Intestinal Homeostasis and Inflammation

Microbial tryptophan catabolites, such as indole and indole derivatives, are potent bioactive molecules that sustain the intestinal barrier’s integrity and contribute to the establishment of immunotolerance against commensal microbes, thereby supporting host–microbiome symbiosis. Bacterial indole derivatives have been largely studied in the context of inflammatory conditions, such as enteric infections or chronic inflammation of the gastrointestinal tract, where their physiological role becomes more manifest.

For instance, the mucin-utilizing bacterium *Peptostreptococcus russellii*, which harbors a gene cluster enabling the production of indoleacrylic acid, has been shown to suppress mucosal inflammation and mitigate epithelial injury upon exposure to dextran sodium sulfate (DSS) in mice [86]. Another example of the benefits of microbial tryptophan digestion is given by the commensal bacterium *Lactobacillus reuteri,* which was found to expand in conditions of unrestricted tryptophan availability (due to genetic ablation of *Ido1* or administration of tryptophan at high concentrations) in the murine stomach and produce indole-3-aldehyde, resulting in AhR-driven IL-22 expression. This IAld–AhR–IL-22 axis was further shown to provide resistance to fungal infection by *Candida albicans*, as well as protection against mucosal inflammation and damage [56]. Furthermore, *L. reuteri* can produce indole-3-aldehyde and indole-3-lactic acid, which activate AhR in ILC3 and intraepithelial CD4^+^CD8αα^+^ double-positive (DP) T cells, respectively [65]. These cells are immunoregulatory T cells that promote oral tolerance and originate from lamina propria CD4 T cells [107]. In mice from different vivaria, the abundance of *L. reuteri* is correlated with the number of these intraepithelial DP T cells and, more importantly, colonization with *L. reuteri* drives the generation of DP T cells through the generation of indole metabolites that activate AhR in intestinal CD4 T cells [65]. Consistent with a protective effect, another *Lactobacillus* sp. (*L. bulgaricus* OLL1181) was found to be capable of activating the AhR signaling in intestinal epithelial cells. Indeed, administration of *L. bulgaricus* OLL1181 to mice induced the expression of the AhR target gene *Cyp1a1* and ameliorated DSS-induced colitis [125]. Furthermore, administration of three *Lactobacillus* strains (*L. murinus* CNCM I-5020, *L. reuteri* CNCM I-5022, and *L. taiwanensis* CNCM I-5019) capable of degrading tryptophan to colitis-susceptible *Card9* (caspase recruitment domain family member 9)-knockout mice attenuates colitis and rescues the mucosal expression of *Il22*, and of its target genes *Reg3b* and *Reg3g,* in an AhR-dependent manner. Importantly, the same study showed that the IBD susceptibility gene *Card9* controls the levels of indigenous microbes and their ability to produce indole derivatives; indeed, the microbiota of *Card9*-/- mice displayed a decrease in *Adlercreutzia* (genus), Actinobacteria (phylum), and *Lactobacillus reuteri* as compared to wild-type mice, as well as impaired production of indole-3-acetic acid. These effects are accompanied by a reduction in intestinal IL-22 expression and the lamina propria ILC3 count. Moreover, transfer of the *Card9*-/- microbiota to germ-free mice leads to increased susceptibility to colitis and lower levels of IL-22, indicating that the unbalanced microbiota drives the immune dysregulation [120]. This study gives an example of the intricate crosstalk between the host and the gut microbiome, and the creation of a vicious cycle in disease where host defects alter the microbiota function which, in turn, contributes to the disease severity. 

In addition to the AhR-dependent effects of microbial tryptophan catabolites on gut mucosal integrity, indole-3-propionic acid (IPA) has been shown to fortify barrier integrity via upregulation of tight-junction molecules, by acting as a ligand for the xenobiotic sensor pregnane X receptor (PXR) in IECs [126]. The importance of IPA-mediated gut barrier integrity has further been proven in a subsequent in vivo study employing the IPA-producing gut symbiont *Clostridium sporogenes*. Colonization of germ-free mice with wild-type *C. sporogenes* resulted in IPA serum concentrations of approximately 80 μM, whereas colonization with a genetically modified form lacking the intact *fldC* subunit of the heterotrimeric enzyme phenyllactate dehydratase (necessary for IPA production) resulted in undetectable levels of IPA in serum and the intestinal lumen, and to a marked increase in gut permeability to FITC–dextran as compared to wild-type-colonized mice. This loss of gut barrier function is consistent with global changes in the host immune profile, including increased proportions of neutrophils, classical monocytes, and activated effector/memory CD4 and CD8 T cells [63].

Interestingly, a recent study demonstrated that the microbial indole derivative indole-3-carboxaldehyde modulates the colonic cellular composition during aging, as administration of indole-3-carboxaldehyde to mice promoted intestinal stem cell turnover, enhanced the proportion of goblet cells and, most importantly, rescued the loss of goblet cells in geriatric mice, in an AhR- and IL-10-dependent manner [127]. Strikingly, the protective functions of indoles on the intestinal barrier are seen even in cases of extreme injury caused by irradiation; indeed, indole 3-propionic acid and indole-3-carboxaldehyde have been shown to facilitate gastrointestinal recovery and increase the survival rate in mice following irradiation [128,129]. 

Collectively, most studies support a beneficial and anti-inflammatory function of AhR; however, some pro-inflammatory effects of AhR have been documented. For instance, in Caco-2 intestinal cells, indole has been found to act as an AhR antagonist at concentrations of 100–250 μM [130]. Microbial and dietary oxazoles were shown to induce IDO1 activity and AhR activation but, instead of promoting tolerance, the oxazole–IDO1–AhR axis triggered natural killer T-cell-dependent intestinal inflammation by modulating lipid antigen presentation by IECs and suppressing IL-10 production [131]. In addition, AhR activation has been shown to polarize T cells towards pathogenic Th17 cells in extraintestinal tissues [101,132,133]. Thus, by sensing exogenous and endogenous molecules, AhR performs multifaceted functions depending on specific ligands and converging signaling from microenvironmental cytokines and factors. 

Although the specific roles of many indole derivatives are not yet fully characterized, fine-tuning the microbial tryptophan metabolism holds promise for therapeutic targets in intestinal inflammatory conditions, provided that future investigations can unravel specific gut strains with indole-producing capacity and the bacteria gene clusters necessary for the enzymatic reactions in the generation of indole products.

## 6. Tryptophan Catabolites in Metabolic Disorders 

Metabolic disorders encompass a cluster of interrelated pathological disorders (e.g., obesity, dyslipidemia, non-alcoholic steatohepatitis, glucose intolerance, insulin resistance, hypertension, and diabetes) that together enhance the risk of cardiovascular diseases (CVDs) and mortality. The state of gut microbiota dysfunction (with loss of microbiota species richness and diversity, restriction of SCFA producers, and increased intestinal permeability) that accompanies metabolic disorders has received increasing attention in the microbiota research field, owing to early pioneer studies establishing a causal relationship between aberrant microbiota composition and metabolic alterations [4]. Indeed, both preclinical and clinical studies employing fecal microbiota transplantations have shown that intestinal engraftment of a healthy microbiota can rescue—at least in part—the metabolic impairments in metabolic syndrome [134,135,136,137]. Similarly, high gene richness and species diversity of human gut microbiota have been associated with metabolic health [138,139,140].

Microbial perturbations and disease severity can reciprocally impact one another. For instance, whereas perturbations of the microbial taxonomic and functional profiles precede the onset of diabetes, hyperglycemia has been shown to provoke intestinal permeability, which can affect the mucosal immunoreactivity towards gut microbes [141,142]. The repertoire of microbiota-derived metabolites act as “exogenous” endocrine signals in the regulation of host metabolism; this is particularly true for the SCFA butyrate, which has been shown to act as an insulin secretagogue, insulin sensitizer, and anti-adipogenic microbial metabolite [143,144,145,146].

In this section, we describe the main findings of studies reporting on the linkage and role of tryptophan catabolites in metabolic health and metabolic disorders.

Similarly to IBD, data from cross-sectional studies indicate that host tryptophan catabolism is altered in individuals with obesity and metabolic syndrome (Table 1). Particularly, the kynurenine-to-tryptophan ratios of blood concentrations are significantly elevated in obese subjects, as well as patients with metabolic syndrome or hyperuricemia, as compared to healthy controls. Moreover, the kynurenine/tryptophan ratios are positively correlated with BMI, as well as triglyceride and uric acid levels, and are effective in stratifying the patient group based on risk of cardiovascular disease [76]. Similarly in a diabetic cohort, kynurenine levels were found to be positively associated not only with BMI, but also with higher HOMA2 insulin resistance index. Moreover, the expression of several enzymes of the kynurenine pathway, including IDO1, was shown to be upregulated in omental adipose tissue of obese women as compared to lean subjects [147]. A recent study analyzed the alterations in plasma and fecal levels of tryptophan catabolites and found that, in line with previous studies, plasma kynurenine levels were higher in obese or type 2 diabetes subjects than in healthy controls, whereas in feces the authors observed a shift of tryptophan catabolism towards more kynurenine and less indole-3-acetic acid production in obese and (non-treated) diabetic patients [148]. These findings suggest an increased activity of intestinal IDO1 and a concomitant inhibition of the microbial indole pathway in the context of metabolic syndrome, similar to the findings on tryptophan metabolism in IBD. 

Overall, high concentrations of kynurenine in plasma and feces are associated with an adverse metabolic profile (i.e., high body weight, fat mass, triglycerides, insulin resistance, and low HDL cholesterol) in obesity [148]. In contrast, fecal levels of microbiota-derived indole, indole-3-acetic acid, 3-methyl-indole, and tryptamine are diminished in obese or diabetic individuals, and are significantly correlated with BMI [149]. Accordingly, untargeted metabolomics analysis of serum metabolites in a prospective cohort study found that microbially produced indole-3-proprionic acid is associated with improved insulin secretion and sensitivity, and with significantly lower risk of developing type 2 diabetes. This association was further corroborated in two other independent population studies [150]. Curiously, another study found that the blood levels of indole-3-propionic acid in obese diabetic patients are lower compared to those in lean individuals, but increase in the three months following gastric bypass surgery, while the circulating levels of tryptophan and kynurenine post-surgery are unchanged [94]. This may indicate that bariatric surgery can improve not only the obesity-related metabolic disorder in patients, but also the metabolic capacity of the microbiome.

Interestingly, mice fed with a HFD (high-fat diet) not only display reduced circulating rates of indole derivatives, but also show a lower copy number of the tryptophanase gene in their microbial genome, indicating a defective microbiota metabolism. Moreover, monocolonization of antibiotic-treated mice with a parental *Escherichia coli* strain or with an *E. coli* strain lacking the *tnaA* gene proves the pivotal role of bacterial tryptophan metabolism in metabolic health, since colonization with the knockout strain aggravates the obesogenic phenotype (with substantially more weight gain and glucose intolerance) [156]. Accordingly, a separate study revealed that dietary intake of bacterial indole-3-priopionic acid reduces fasting glucose, fasting insulin, and the HOMA insulin resistance index in rats, substantiating the clinical findings of the positive association between IPA and insulin sensitivity [150,157]. Furthermore, preclinical models show the therapeutic potential of targeting the shift in tryptophan metabolism in metabolic syndrome. In fact, HFD-fed mice and leptin-deficient ob/ob mice display a microbiota with impaired metabolic production of AhR ligands—such as indole, indole-3-acetic acid, and tryptamine—and, accordingly, lower intestinal expression of *Il22*. However, in vivo treatment with the AhR agonist Ficz alleviates the metabolic impairments (e.g., insulin and glucose dysmetabolism, intrahepatic lipid accumulation, and dyslipidemia) in both diet- and genetic-induced metabolic syndrome. Similarly, administration of the commensal strain *Lactobacillus reuteri,* which exhibits high AhR-ligand production, improves glucose clearance, insulin sensitivity, and liver steatosis in HFD-fed mice, whereas administration of a non-indole-producing *Lactobacillus* strain does not rescue the HFD-induced dysregulation of glucose metabolism. Mechanistically, forcing AhR signaling with Ficz ameliorates the intestinal barrier dysfunction and accompanied systemic inflammation in obese mice, and can stimulate the secretion of glucagon-like protein 1 (GLP-1) in enteroendocrine L cells in vitro [149]. In this regard, the incretin hormone GLP-1 is produced by intestinal enteroendocrine cells in response to dietary nutrients as well as specific microbial metabolites; it plays a crucial role in metabolism by stimulating insulin secretion by pancreatic beta cells, promoting fat oxidation and energy expenditure, while suppressing appetite [158]. A second study revealed that targeting the shift in tryptophan catabolism by suppressing host IDO1 activity also promotes metabolic health in preclinical models of metabolic syndrome. Indeed, genetic ablation of *Ido1* protects mice from HFD-induced adiposity, insulin resistance, steatohepatitis, and macrophage infiltration of liver and adipose tissue. Likewise, pharmacological inhibition of IDO1 activity with L-1methyl tryptophan (1MT) ameliorates insulin resistance upon HFD-feeding or in genetically obese ob/ob mice. Mechanistically, these beneficial effects, in the absence of IDO1 activity, are due to changes in the microbiota profile, with lower proportions of *Clostridiales*—in particular *Lachnospiraceae*—and rewiring of tryptophan metabolism towards the microbial indole pathway, as attested by enhanced intestinal indole-3-acetic acid in Ido1-/- or 1MT-treated obese mice, and by the beneficial effects of transferring the microbiota of 1MT-treated mice into obese ob/ob mice. Moreover, IDO-1 deficiency restores the intestinal barrier function and IL-22 expression, which are impaired in obesity. Blocking the IAA–AhR–IL-22 axis by treatment with neutralizing anti-IL-22 antibodies abrogates the protective effects of IDO1 deficiency against insulin sensitivity, liver steatosis, and intestinal permeability [148]. These preclinical studies underscore the importance of microbial tryptophan catabolism and, hence, microbiota-mediated AhR signaling, in the promotion of metabolic health. Notably, these findings are supported by human data showing that low AhR signaling is correlated with enhanced inflammatory tone in the jejunum epithelium of obese individuals [159]. Moreover, IL-22 was previously found to reverse metabolic disturbances in obese, leptin-receptor-deficient db/db mice, as well as improving intestinal barrier function and endotoxemia [160]. Despite the clear benefits of blocking the kynurenine pathway in the context of obesity and metabolic syndrome, another study revealed that in mice fed a conventional diet, kynurenine, when administered daily for 7 days, increases energy expenditure while reducing subcutaneous and visceral adiposity. Particularly in white adipose tissue, kynurenine administration triggers the upregulation of genes involved in the thermogenesis program, fatty acid oxidation, and oxidative phosphorylation, together with the induction of an immunoregulatory gene signature associated with Treg cells and ILC2s. Furthermore, a 2-week treatment with kynurenine prevented weight gain in HFD-fed mice, and also improved glucose clearance and reduces subcutaneous fat mass and triglyceride levels [72]. These data are in contrast with the observation that administration to IDO1-deficient mice did not change their body weight, fat mass, or insulin sensitivity [148]; these contrasting results may be explained by differences in genetic background and supraphysiological concentrations of kynurenine administered by daily intraperitoneal gavage.

In support of the protective effects of microbial tryptophan catabolites in metabolic disorders, the indole metabolite has been shown to instigate GLP-1 secretion by immortalized and primary murine colonic enteroendocrine L cells. Curiously, the beneficial effects of indole on L-cell function exhibit a biphasic temporal response—while short-term exposure (6 min) induces calcium entry and acute GLP-1 secretion, prolonged exposure (240 min) slows ATP production, blunting the release of GLP-1 [161]. Overall, these data are consistent with the above-described positive effect of AhR activation on GLP-1 secretion by L cells [149]. Protective effects of indole have also been found in the liver; indeed, indole was shown to alleviate the LPS-induced upregulation of pro-inflammatory cytokines and alterations in cholesterol metabolism in murine livers [149]. Accordingly, a recent study revealed that oral administration of physiological concentrations of indole to genetically obese ob/ob mice reduces hepatic damage and inflammation, with lower cytokine production and macrophage activation in the liver, but does not prevent hepatosteatosis [162]. Unexpectedly, administration of indole to mice, upon HFD-feeding, ameliorated glucose clearance and the severity of obesity by negatively regulating the expression of the microRNA miR-181 which, by tuning the gene expression program, contributes to insulin resistance and inflammation in white adipose tissue in obesity [156]. 

Interestingly, blocking the serotonin pathway in tryptophan metabolism results in metabolic benefits similar to those observed in the absence of IDO1 activity. Indeed, genetic *Thp1* ablation or pharmacological inhibition of THP1 protects against obesity, insulin resistance, and fatty liver disease in mice fed an HFD, while increasing the overall metabolic expenditure. Particularly, the absence of THP1 significantly increases the metabolic activity of the brown adipose tissue due to the β–adrenergic induction of thermogenesis, which is blunted in the presence of serotonin [163]. These data are partially consistent with the observation that specific abrogation of intestinal serotonin production, by gut-specific *Thp1* deletion, restores both insulin and glucose tolerance as well as lipolysis and gluconeogenesis in HFD-induced metabolic syndrome; however, the effects on energy expenditure and thermogenesis were not investigated in this study [164]. Considering that the great majority of serotonin is produced in gut enterochromaffin cells, it is not surprising that total-body *Thp1*-knockout mice show a similar phenotype to that of mice with a gut-specific deletion. These findings on serotonin deficiency in preclinical models are supported by human data, as polymorphism in THP1 has been associated with obesity traits—such as waist circumference and BMI—and elevated plasma levels of the serotonin end-product 5-hydroxyindole-3-acetic acid (5-HIAA) have been reported in patients with metabolic syndrome [151,165]. In particular, the plasma concentrations of 5-HIAA are associated with a deleterious plasma metabolic profile, being positively correlated with fasting glucose and triglycerides, and negatively correlated with HDL cholesterol [151]. Furthermore, serotonin signaling appears to be essential for proper maintenance of energy sources (e.g., glycerol, free fatty acids, and ketone bodies) during fasting periods. Indeed, the utilization of gut-specific *Thp1*-knockout mice revealed that enterochromaffin-cell-derived serotonin stimulates lipolysis in adipocytes and gluconeogenesis in hepatocytes, and simultaneously blocks glucose uptake by hepatocytes during fasting. As a result of these effects, in the absence of gut-derived serotonin, the circulating levels of energetic substrates were blunted during fasting, but insulin sensitivity and glucose clearance were improved upon insulin and glucose challenge, respectively [164].

Collectively, these studies suggest that all three tryptophan degradation pathways are essential in metabolic homeostasis, and that the aberrant activation of host degradation pathways at the expense of microbial tryptophan degradation and production of indole metabolites contributes to metabolic impairment in obesity and diabetes.

## 7. Tryptophan Catabolites in Chronic Kidney Diseases and Cardiovascular Diseases

As for other distal organs, accumulating evidence underscores a bidirectional crosstalk between microbiota and kidneys, which becomes particularly evident in progressive chronic kidney diseases. Indeed, CKD is associated with intestinal dysbiosis and accumulation of microbiota-derived uremic toxins, including indoxyl sulfate. In CKD, a deleterious vicious cycle has been postulated to drive dysbiosis, as well as kidney damage. In fact, the decline in renal function leads to systemic accumulation of uremic toxins, thereby promoting the transfer of urea and other waste products to the gut. In particular, the distal intestine becomes a major route of excretion in CKD. The resulting accumulation of urea in the gut increases the luminal pH, which negatively affects the intestinal barrier integrity as well as the microbial ecosystem [7,166]. On the other hand, the accumulation of uremic toxins in the intestinal lumen, restriction in fiber intake (due to the nutritional management of CKD patients to limit their potassium intake), and the CKD-associated increase in transit time [167,168,169,170,171,172] result in a selective pressure on the gut microbial communities that favors the outgrowth of bacteria harboring urease and uricase activities, and a shift towards proteolytic bacteria at the expense of saccharolytic microbes [53,173,174,175].

Prototypic microbiota-dependent uremic toxins of protein origin include indoxyl sulfate (IS), p-cresol sulfate (PCS), trimethylamine N-oxide (TMAO), and phenylacetylglutamine (PAG) [29,32,176]. Although indole derivatives generally exhibit beneficial effects on the host physiology, IS has been implicated in the pathogenesis of CKD. Indoxyl sulfate is generated through hepatic conversion of indole by action of the cytochrome P450 enzymes and sulfotransferase; IS reaches serum concentrations of 100 μM in CKD patients, as opposed to typical levels of 2 μM in healthy subjects [31,152,177].

Multiple reports have associated the levels of microbiota-derived uremic toxins with higher risks of mortality, cardiovascular events, and progression of renal disease in CKD patients [178]. For instance, a meta-analysis utilizing data from 11 studies showed that increases in circulating IS and PSC were associated with increased mortality in subjects with CKD patients [153]. Similarly, increased IS concentrations in hemodialysis patients were associated with all-cause mortality and with first heart failure events, whereas the combined increase in the concentrations of four uremic toxins was correlated with higher markers of renal dysfunction, creatinine and urine, and increased risk of cardiovascular mortality [154,179,180]. Furthermore, a recent study proved a causal role of aberrant microbiota in the progression of renal disease by transplanting the feces of CKD patients into germ-free or antibiotic-treated rats, which consequently displayed higher serum concentrations of uremic toxins and an augmentation of oxidative stress and fibrosis in the kidneys. Moreover, intestinal colonization with only two species identified in the microbiomes of patients with end-stage renal disease—*Eggerthella lenta* and *Fusobacterium nucleatum*—drove the production of uremic toxins and aggravated renal disease in a rat model of CKD [181]. Despite being an AhR ligand [182], multiple studies indicate a harmful effect of IS on kidney cells; in proximal tubular epithelial cells, indoxyl sulfate was reported to provoke oxidative stress and induce an inflammatory and fibrotic phenotype [183,184]. Similarly, in endothelial cells, IS triggered oxidative stress and senescence, while inhibiting proliferation [174,185,186,187,188]. In CKD patients, the systemic concentrations of IS were found to be correlated with diminished rates of flow-mediated endothelium-dependent vasodilatation [187].

Importantly, the microbial production of indole can be a modifiable target in CKD; indeed, an early study reported the feasibility of genetically manipulating the indole producer *Bacteroides thetaiotaomicron (Bt)* to delete the tryptophanase gene. Additionally, monocolonization of wild-type and mutant *Bt* into germ-free mice resulted in robust and undetectable urinary levels of IS, respectively; similar results were obtained when the gut colonization occurred in the context of a synthetic community of six bacteria [85]. A more recent study found that a diet rich in sulfur amino acids inhibits microbial tryptophanase activity via post-translational modification involving S-sulfhydration. The study further revealed that the high-sulfur-amino-acid diet abrogated the toxic effects of IS on the kidneys, as it diminished serum creatinine levels and alleviated renal injury—including tubular dilatation, tubulitis, and fibrosis—in an adenine-induced murine model of CKD [189].

Finally, IS is harmful not only to the kidneys, but also to the vasculature. In fact, administration of IS to hypertensive rats was found to trigger aortic calcification with expression of osteoblast differentiation markers, and to cause wall thickening in the arcuate, thoracic, and abdominal aorta [190]. In addition, serum IS levels were positively correlated with aortic calcification and arterial stiffness in CKD patients [155]. Furthermore, IS acts as a pro-thrombotic factor by inducing tissue factors in an AhR-dependent manner in vascular smooth muscle cells and endothelial cells [155,191]. Similarly, in the context of coronary disease, the plasma levels of the AhR ligand kynurenine are associated with worse cardiovascular outcomes in patients with acute myocardial infarction. Moreover, unstable atheromatous plaques contain high levels of kynurenine, in contrast to the undetectable concentrations in stable fibrous plaques [71]. Additionally, in atherosclerosis-prone low-density-lipoprotein-receptor-deficient (*Ldlr*−/−) mice, IDO1 deficiency counteracts the development of atherosclerosis. Mechanistically, IDO1 activity in macrophages drives atherosclerosis and increased plaque size by generating kynurenine which, in turn, inhibits the production of the anti-inflammatory cytokine IL-10. Indeed, the protective effects of *Ido1* ablation are blunted in the absence of *Il10*, or upon supplementation with kynurenine in vivo [71].

Overall, these findings in the context of CKD or CVD indicate that excessive accumulation of tryptophan catabolites is deleterious and associated with poor health outcomes in both diseases (Table 1). As discussed above, AhR activation can have contrasting effects on inflammation, possibly depending on the ligand origin and concentration, the cell type, and other microenvironmental signals.

## 8. Future Directions

It is clear that tryptophan catabolites impact several aspects of host physiology and, thus, modulate the severity and outcomes of pathologies affecting distinct host compartments. However, further studies are warranted to untangle which gut bacteria are the most prominent producers of indoles in specific diseases, and to dissect which microbial enzymes catalyze the rate-limiting steps in the generation of indole derivatives. For this purpose, metagenomics analyses of stool samples from human cohorts, together with bacterial metabolome studies, are necessary to advance precision medicine targeting specific commensal species and/or enzymatic reactions. Indeed, in addition to such association studies, there is an urgent need for more mechanistic insights into the exact roles and modes of action of microbial metabolites in host (patho)physiology in order to advance precise therapeutic targeting. Finally, we cannot forget that the host–gut microorganism interactions are mutualistic and bidirectional; hence, metabolic profiling of stool, urine, and serum specimens may provide a more comprehensive view of the fates of endogenous or dietary substrates in healthy and diseased subjects, as attested by the (herein) described disequilibrium between microbial and host-mediated degradation of tryptophan in metabolic and inflammatory human disorders.

## Figures and Tables

**Table 1:** Altered levels of tryptophan-derived metabolites and associations with clinical outcomes in metabolic, cardiovascular, and chronic kidney diseases.

**Metabolic Disorders**			
**Metabolite**	**Increased/Decreased/Association**	**Patient Group**	**Reference**
Kynurenine/tryptophan ratio	Increased	Obese/metabolic syndrome	[76]
Kynurenine	+ associated with BMI, HOMA-index	Diabetes	[147]
Kynurenine	Increased	Type 2 diabetes/obese	[148]
Indole	Decreased (feces)	Type 2 diabetes/obese	[149]
Indole-3 acetic acid	Decreased (feces)	Type 2 diabetes/obese	[149]
3-methyl-indole	Decreased (feces)	Type 2 diabetes/obese	[149]
Tryptamine	Decreased (feces)	Type 2 diabetes/obese	[149]
Indole-3 propionic acid	+ associated with insulin sensitivity	Type 2 diabetes	[150]
Indole-3 propionic acid	Decreased	Type 2 diabetes/obese	[94]
5-hydroxyindole-3-acetic acid	Increased	Metabolic syndrome	[151]
**Chronic Kidney and Cardiovascular Diseases**			
**Metabolite**	**Increased/Decreased/Association**	**Patient Group**	**Reference**
Indoxyl sulfate	Increased	CKD, ESRD	[32,152]
Indoxyl sulfate	+ associated with mortality	CKD	[153]
Indoxyl sulfate	+ associated with hearth failure	CKD hemodyalysis	[154]
Indoxyl sulfate	+ associated with aortic calcification	CKD	[155]
Indoxyl sulfate	+ associated with arterial stiffness	CKD	[155]
Kynurenine	+ associated with myocardial infarction	Coronary artery disease	[71]
Kynurenine	Increased (in unstable atherosclerotic plaques)	Coronary artery disease	[71]
